# Deep Cliques in Point Sets

**DOI:** 10.1007/s00454-023-00612-y

**Published:** 2023-12-18

**Authors:** Stefan Langerman, Marcelo Mydlarz, Emo Welzl

**Affiliations:** 1 Département d’informatique, Université Libre de Bruxelles, Brussels, Belgium; 2 Universidad Nacional de General Sarmiento and CONICET, Buenos Aires, Argentina; 3 Department of Computer Science, ETH Zürich, 8092 Zürich, Switzerland

**Keywords:** *k*-Sets, *j*-Facets, Halving lines, Cliques, Depth measures, Tukey depth, *k*-Degenerate graphs, 05C35, 05D99, 52C35, 52C45, 52C10, 68U05

## Abstract

Let $n \in \mathbb {N}$ and $k \in \mathbb {N}_0$. Given a set *P* of *n* points in the plane, a pair $\{p,q\}$ of points in *P* is called *k*-*deep*, if there are at least *k* points from *P* strictly on each side of the line spanned by *p* and *q*. A *k*-*deep clique* is a subset of *P* with all its pairs *k*-*deep*. We show that if *P* is in general position (i.e., no three points on a line), there is a *k*-deep clique of size at least $\max \{1,\lfloor \frac{n}{k+1} \rfloor \}$; this is tight, for example in convex position. A *k*-deep clique in any set *P* of *n* points cannot have size exceeding $n-\lceil \frac{3k}{2} \rceil$; this is tight for $k \le \frac{n}{3}$. Moreover, for $k \le \lfloor \frac{n}{2} \rfloor - 1$, a *k*-deep clique cannot have size exceeding $2\sqrt{n(\lfloor \frac{n}{2} \rfloor -k)}$; this is tight within a constant factor. We also pay special attention to $(\frac{n}{2}-1)$-deep cliques (for *n* even), which are called *halving cliques*. These have been considered in the literature by Khovanova and Yang, 2012, and they play a role in the latter bound above. Every set *P* in general position with a halving clique *Q* of size *m* must have at least $\lfloor \frac{(m-1)(m+3)}{2}\rfloor$ points. If *Q* is in convex position, the set *P* must have size at least $m(m-1)$. This is tight, i.e., there are sets $Q_m$ of *m* points in convex position which can be extended to a set of $m(m-1)$ points where $Q_m$ is a halving clique. Interestingly, this is not the case for all sets *Q* in convex position (even if parallel connecting lines among point pairs in *Q* are excluded).

## Introduction

Throughout this paper *P* is a finite set of points in the plane $\mathbb {R}^2$. We say that *P* is in *general position* if no three points in *P* lie on a common line. We start with the key notion of this paper, which relates to Tukey depth from statistics.^1^

### Definition 1.1

(*k*-*deep pair*) A pair  is called *k*-*deep* if at least *k* points from *P* lie strictly on each side of the line spanned by *p* and *q*. Let $\textsf{G}_{k}\hspace{-0.05em}(P)$ be the graph with vertex set *P* and the *k*-deep pairs of *P* as its edges. A clique in $\textsf{G}_{k}\hspace{-0.05em}(P)$ is called a *k*-*deep clique*.

Answering a question asked by Ferran Hurtado in 2011^2^ we show the following result.

### Theorem 1.2

Let *P* be a set of *n* points, $n \in \mathbb {N}$. For $k \in \mathbb {N}_0$, the following holds:

(i) If *P* is in general position, then there is a *k*-deep clique of size at least $\max \{1,\lfloor \frac{n}{k+1} \rfloor \}$. The bound is attained for *P* in convex position.
(ii) A *k*-deep clique cannot have size exceeding $n-\lceil \frac{3k}{2} \rceil$. This bound is tight provided $k \le \frac{n}{3}$.
(iii) For $k \le \lfloor \frac{n}{2} \rfloor - 1$, a *k*-deep clique cannot have size exceeding $2\sqrt{n(\lfloor \frac{n}{2} \rfloor -k)}$; this is tight within a constant factor.

**Fig. 1:**
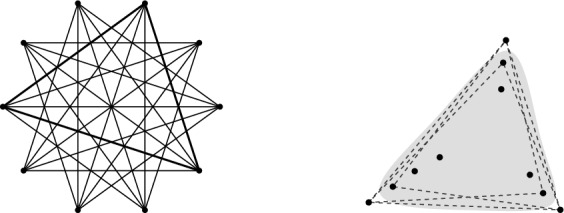
Examples of *k*-deep cliques, for $k=2$, in sets of $n=10$ points. Left: A set $P^\textsf{c}$ in convex position, with the graph $\textsf{G}_{2}\hspace{-0.05em}(P^\textsf{c})$ of 2-deep pairs, and a maximum clique of size $\lfloor \frac{n}{k+1} \rfloor = \lfloor \frac{10}{3} \rfloor = 3$. Right: A set *P* with the dashed edges of the graph $\overline{\textsf{G}}_{2}\hspace{-0.05em}(P)$ complementary to $\textsf{G}_{2}\hspace{-0.05em}(P)$; the shaded area covers a clique of size $n-\lceil \frac{3k}{2} \rceil = 10 - \lceil \frac{3\cdot 2}{2} \rceil = 7$ in $\textsf{G}_{2}\hspace{-0.05em}(P)$

The fact that the bound in (i) is tight for sets $P^\textsf{c}$ in convex position is easily seen, see Fig. 1  (left). A set *Q* of vertices in $\textsf{G}_{k}\hspace{-0.05em}(P^\textsf{c})$ forms a clique if and only if any $k+1$ consecutive points in the cyclic order along the boundary of the convex hull of $P^\textsf{c}$ contain at most one point in *Q*. This shows $|Q| \le \lfloor \frac{n}{k+1} \rfloor$, unless $n \le k$.

The proof of the lower bound in Theorem 1.2(i) is given in Sect. 2, the upper bound in Theorem 1.2(ii) is shown in Sect. 3. In Sect. 5 we derive Theorem 1.2(iii), which gives bounds relevant for *k* between $\frac{n}{3}$ and $\frac{n}{2}$. These bounds are tight within a constant factor. Note that the upper bound in Theorem 1.2(iii) is better than the one in Theorem 1.2(ii) when $k > \frac{2(\sqrt{10}-1)}{9} n \approx 0.48 n$.

To obtain this last result, we study in Sect. 4 the extreme case of $(\frac{n}{2}-1)$-deep cliques, for *n* even, which is of independent interest. We call these also halving cliques, since we require here that every connecting line of the clique has the same number of points on both sides. Such halving cliques have been previously investigated by Khovanova and Yang [9, 10], and we sharpen the picture for those.

### Theorem 1.3

Let *Q* be a halving clique of size *m* in a set of *n* points in general position.

(i) $$n \ge \lfloor \frac{(m-1)(m+3)}{2} \rfloor $$.
(ii) If *Q* is in convex position, then $n \ge m(m-1)$. This bound is tight.

The bound in (i) can be read as $m \le \sqrt{2n+4}-1$ which is a slight improvement over a bound of $m \le \sqrt{2n}+1$ in [9]. The improved bound is tight for $m \le 5$, but we do not expect it to be tight for large *m*. More interestingly, the tightness construction for (ii) implies that there are *n* points with a halving clique of size $\lfloor \sqrt{n} \rfloor$ which improves over a construction with a halving clique of size $\sqrt{\frac{n}{2}}$ in [9]. The essential insight here is that when it comes to extending a set *Q* in convex position to a smallest possible set *P* where *Q* is a halving clique, it is relevant how the pairwise connecting lines arrangement of *Q* looks like.

For the context where these investigations reside, let us mention that the space of all possible dissections of a planar point set by lines, and how the lines dissect with respect to the number of points on each side, has been a topic of interest since papers by Lovász [11] and Erdös et al. [6] in the early seventies, among others with the Goodman-Pollack characterization [8] of order types by so-called $\lambda$-matrices (which list for each ordered pair of points the number of points to the left of the directed line through these two points). One of the main problems in the area is to find a tight bound on the number of *k*-sets (the number of dissections with *k* points on one side), the number of *k*-edges (see Definition 1.4 below), and in particular the number of halving lines. The current best upper bound of $O(nk^{1/3})$ was proved by Dey [4] while the best known lower bound construction of $n e^{\Omega (\sqrt{\log {k}})}$ was shown by Tóth [14], see [16] for a survey.

In this section we still recall *j*-edges (related to *k*-sets) of point sets, see e.g., [7, 16], and *k*-degeneracy of graphs, since these will be needed in our proofs.

### *j*-Edges in Point Sets

#### Definition 1.4

(*j*-*edge*) An ordered pair *pq* of two distinct points in *P* is called a *j*-*edge* if there are exactly *j* points from *P* strictly to the left of the line through *p* and *q*, directed from *p* to *q*. *pq* is called a $(\le k)$-*edge* (a $(\ge k)$-*edge*, resp.), if it is a *j*-edge with $j \le k$ (with $j\ge k$, resp.).

Note that if *P* is in general position, *pq* is a *j*-edge if and only if *qp* is an $(n-j-2)$-edge. By definition, an unordered pair $\{p,q\}$ of points in *P* is *k*-deep if and only if both *pq* and *qp* are $(\ge k)$-edges.

Rather than looking at cliques in the graph of *k*-deep pairs, we will consider the equivalent question about independent sets in its complementary graph.

#### Definition 1.5

(*k*-*shallow pair*) A pair $\{p,q\}$ of points in *P* is called *k*-*shallow* if it is not *k*-deep. Let $\overline{\textsf{G}}_{k}\hspace{-0.05em}(P)$ be the graph with vertex set *P* and with the *k*-shallow pairs of *P* as its edges. ($\overline{\textsf{G}}_{k}\hspace{-0.05em}(P)$ is the graph complementary to the graph $\textsf{G}_{k}\hspace{-0.05em}(P)$ of *k*-deep pairs.)

A pair $\{p,q\}$ is *k*-shallow if and only if either *pq* or *qp* is a $(\le k-1)$-edge. Note that, provided *P* is in general position, for $k-1 < \frac{n-2}{2}$ ($\Leftrightarrow k < \frac{n}{2}$), at most one of *pq* and *qp* can be a $(\le k-1)$-edge, i.e., the number of *k*-shallow pairs is exactly the number of $(\le k-1)$-edges. The maximum possible number of such $(\le k-1)$-edges in a set of *n* points is known.

#### Theorem 1.6

(Alon and Györi [3]^3^, Peck [13]) If *P* is in general position and $0 \le k < \frac{n}{2}$, the number of $(\le k-1)$-edges is at most *kn*. Convex position attains this in the given range.

This yields a lower bound (of roughly $\frac{n}{2k+1}$) on the size of independent sets in the graph of *k*-shallow pairs via Turán’s Theorem. However, for our geometric setting we will establish a better bound proceeding along (almost) *k*-degenerate graphs, instead.

### *k*-Degenerate Graphs

The following is a standard graph theoretic notion.

#### Definition 1.7

A simple undirected graph $G=(V,E)$ is called *k*-*degenerate* if its vertices can be ordered as $(v_1,v_2,\ldots ,v_n)$, $n:= |V|$, such that for all *i*, $v_i$ has at most *k* neighbors in $\{v_{i+1}, \ldots , v_n\}$.

It is easily seen that every *k*-degenerate graph has a proper coloring with at most $k+1$ colors $\{1,2,\ldots ,k+1\}$: We color the vertices starting with $v_n$ down to $v_1$, and we assign to each $v_i$ the smallest color not appearing among its neighbors in $\{v_{i+1}, \ldots , v_n\}$. The vertices of a given color form an independent set, hence a *k*-degenerate graph always has an independent set of size at least $\lceil \frac{n}{k+1} \rceil$.

Note that Theorem 1.6 shows that the graph $\overline{\textsf{G}}_{k}\hspace{-0.05em}(P)$ of *k*-shallow pairs has average degree at most 2*k*. In fact, any subgraph of $\overline{\textsf{G}}_{k}\hspace{-0.05em}(P)$ induced by a subset *Q* of *P* has average degree at most 2*k*. (In general, graphs may not preserve an upper bound on the average degree in their induced subgraphs. An easy counterexample would be a union of a clique and an independent set.) For that, note that if we have a subset *Q* of *P*, the *Q*-induced subgraph of $\overline{\textsf{G}}_{k}\hspace{-0.05em}(P)$ is a subgraph of $\overline{\textsf{G}}_{k}\hspace{-0.05em}(Q)$, where *k*-shallow is defined with respect to  *Q*, since removing points cannot make a pair less shallow. This shows that the graph of *k*-shallow pairs is 2*k*-degenerate and thus an independent set of size $\lceil \frac{n}{2k+1} \rceil$ must exist. For the improvement of this bound to $\lceil \frac{n}{k+1} \rceil$ we will consider a relaxation of the notion of *k*-degeneracy.

### General vs. Special Position (Geometric Degeneracies)

Let *P* be a point set, possibly with collinearities, and let $\tilde{P}$ be a perturbation of *P* to general position (i.e., all nondegenerate triangles formed by points in *P* preserve orientation). Given a *j*-edge *pq*, the perturbation of *p* and *q* to $p'$ and $q'$, resp., will result in a $j'$-edge $p'q'$ with $j' \ge j$. In other words, a pair of points can only get deeper by perturbation. That is, $\overline{\textsf{G}}_{k}\hspace{-0.05em}(\tilde{P})$ is a subgraph of $\overline{\textsf{G}}_{k}\hspace{-0.05em}(P)$, and $\textsf{G}_{k}\hspace{-0.05em}(P)$ is a subgraph of $\textsf{G}_{k}\hspace{-0.05em}(\tilde{P})$.

On the one hand, lower bounds as in Theorem 1.2(i) are interesting only for general position, since otherwise a set of points on a common line trivializes the problem. On the other hand, when the goal is to exhibit sets with large *k*-deep cliques, we can employ sets in special position (i.e., sets with some collinear triples) – any perturbation of such a set can generate only even larger *k*-deep cliques.

## Lower Bound—Proof of Theorem 1.2(i)

### Almost *k*-Degenerate Graphs

#### Definition 2.1

A simple undirected graph $G=(V,E)$ is called *almost* *k*-*degenerate* if all but *k* of its vertices can be ordered as $(v_{k+1},v_{k+2},\ldots ,v_n)$, $n:= |V|$, such that for all *i*, $v_i$ has at most *k* neighbors in $\{v_{i+1}, \ldots , v_n\}$.

#### Lemma 2.2

Every almost *k*-degenerate graph *G* with *n* vertices has an independent set of size at least $\lfloor \frac{n}{k+1} \rfloor$.

#### Proof

It follows directly from the definition of almost *k*-degenerate, and the preceding observation, that *G* has a subgraph induced by $n-k$ vertices with an independent set of size $\lceil \frac{n-k}{k+1} \rceil = \lfloor \frac{n}{k+1} \rfloor$. Obviously, this set is also independent in the whole graph.

$$\square $$

Hence, all we need in order to prove Theorem 1.2(i) is to show that $\overline{\textsf{G}}_{k}\hspace{-0.05em}(P)$ is almost *k*-degenerate.

### The *j*-Edges Incident to a Point

The following lemma (see, e.g., [7, Lem. 4.2]) follows from a simple rotational argument as given in early papers by Lovász [11] and Erdös et al. [6] on this topic.

#### Lemma 2.3

Let *P* be a set of *n* points in general position, and assume $j, k \in \mathbb {N}_0$, $j \le k-1$. Let *h* be a directed line containing exactly one point *p* in *P*, such that there are at least *k* points on both sides of the line. Then for *p*, the number of incoming *j*-edges *qp* with $q \in P$ left of *h* equals the number of outgoing *j*-edges *pq* with $q \in P$ right of *h*.

**Fig. 2:**
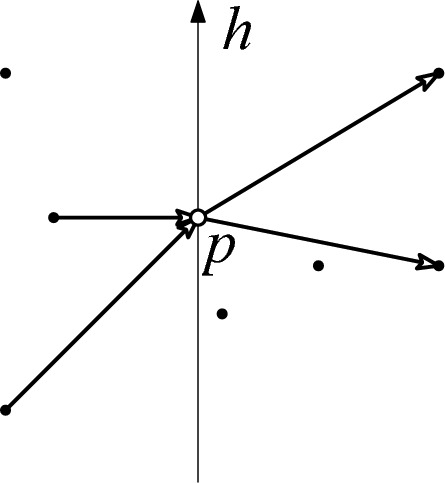
A line *h* through *p* with at least 3 points on both sides. The 2-edges incoming to *p* from left of *h* (2 of them), and the 2-edges outgoing to the right of *h* (2 of them)

#### Lemma 2.4

For *P* in general position, $\overline{\textsf{G}}_{k}\hspace{-0.05em}(P)$ is almost *k*-degenerate.

#### Proof

Let us assume that no two points in *P* have the same *x*-coordinate (if necessary, perform a slight rotation of the set). Let $(p_1,p_2, \ldots , p_n)$ be the order of *P* in increasing *x*-coordinate. We want to show that for every point $p_i$ with $i \ge k+1$, the number of $(\le k-1)$-edges incident to $p_i$ (incoming or outgoing) with the other endpoint in $P_i^+:= \{p_{i+1},p_{i+2},\ldots ,p_n\}$ is at most *k*. This will imply that the order $(p_{k+1},p_{k+2},\ldots ,p_n)$ (with $\{p_1,p_2, \ldots , p_k\}$ from *P* skipped) witnesses the fact that $\overline{\textsf{G}}_{k}\hspace{-0.05em}(P)$ is almost *k*-degenerate.

When considering $p_i$ with $i \ge k+1$, we first observe that the claim is trivial if $|P_i^+| \le k$. Otherwise, let us remove all points $p_{k+1},p_{k+2},\ldots , p_{i-1}$. In the resulting set $P {\setminus } \{p_{k+1},p_{k+2},\ldots , p_{i-1}\}$, $p_i$ can be incident to only more $(\le k-1)$-edges with points in $P_i^+$. For every $j \le k-1$, by Lemma 2.3 (applied on the subset $P {\setminus } \{p_{k+1},p_{k+2},\ldots , p_{i-1}\}$ with *h* the vertical line upwards through $p_i$), the number of *j*-edges outgoing to $P_i^+$ equals the number of incoming *j*-edges from $\{p_1,p_2, \ldots , p_k\}$. Similarly, the number of *j*-edges incoming from $P_i^+$ equals the number of outgoing *j*-edges to $\{p_1,p_2, \ldots , p_k\}$ (note that we assume $|P_i^+| > k$).

Every *k*-shallow pair containing $p_i$ must be, for some orientation and for some $j \le k-1$, either an incoming *j*-edge or an outgoing *j*-edge, not possibly both (because of general position), since $\left| P {\setminus } \{p_{k+1},p_{k+2},\ldots , p_{i-1}\}\right| = k + 1 + |P_i^+| \ge 2(k+1)$. It follows that the number of *k*-shallow pairs incident to *p* and some point in $P_i^+$ equals the number of *k*-shallow pairs incident to *p* and some point in $\{p_1,p_2, \ldots , p_k\}$, which is at most *k*. $\square$

By Lemma 2.2, $\overline{\textsf{G}}_{k}\hspace{-0.05em}(P)$ has an independent set of size $\lfloor \frac{n}{k+1} \rfloor$, therefore its complement $\textsf{G}_{k}\hspace{-0.05em}(P)$ has a clique of that size and Theorem 1.2(i) follows.

## Upper Bound for *k* Small—Proof of Theorem 1.2(ii)

**Fig. 3:**
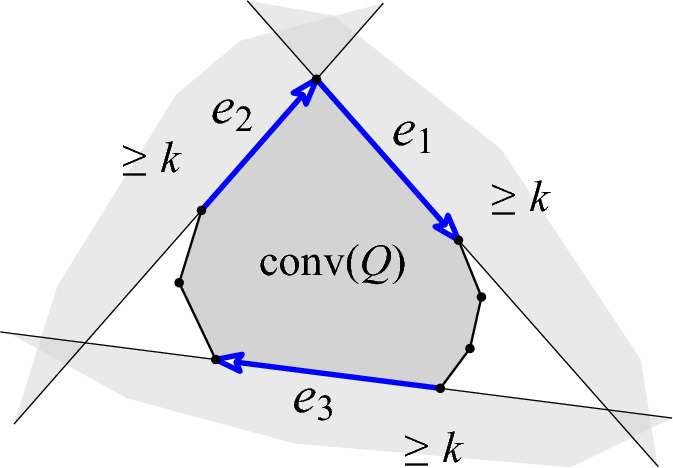
Illustrating the proof of Theorem 1.2(ii)

### Proof

*(of upper bound in Theorem* 1.2(ii), *i.e., a* *k-deep clique cannot have size exceeding* $n-\lceil \frac{3k}{2} \rceil$) Let *Q* be a *k*-deep clique in a set *P*. Choose three directed edges $e_1,e_2,e_3$ of the convex hull of *Q* such that, for each $i=1,2,3$, *Q* lies in the *closed* halfplane $\overline{R}(e_i)$ to the right of the directed line through $e_i$, and, moreover, the intersection of these halfplanes is bounded (Fig. 3). Now let $L(e_i)$ be the *open* halfplane to the left of the directed line through $e_i$. Since the pair of points defining $e_i$ is *k*-deep, $L(e_i)$ contains at least *k* points from *P*. Since $L(e_1) \cap L(e_2) \cap L(e_3)$ is empty (this follows from $\overline{R}(e_1) \cap \overline{R}(e_2) \cap \overline{R}(e_3)$ being bounded), we have $|(L(e_1) \cup L(e_2) \cup L(e_3))\cap P| \ge \frac{3k}{2}$. This gives at least $\lceil \frac{3k}{2} \rceil$ points not in *Q* and concludes the argument. $\square$

**Fig. 4:**
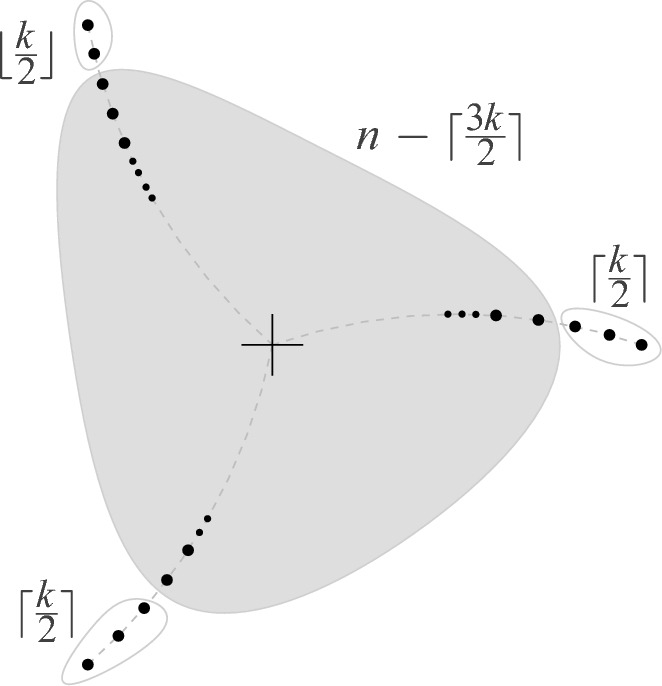
*n* points, $n \ge 3k$, with a *k*-deep clique of size $n-\lceil \frac{3k}{2} \rceil$ (here $k=5$)

### Proof

(*of tightness of the bound in Theorem* 1.2(ii) for $k \le \frac{1}{3}$) Consider three rays emanating from the origin, with no halfplane containing all three rays. Place the *n* points on these three rays so that each ray has at least *k* points. Note that every line connecting two points on the same ray has at least *k* points (on the other rays) on both sides, see Fig.  4. Recall the short discussion about general vs. special position in Sect. 1.3, i.e., we allow here collinear points (a perturbation will preserve *k*-deep cliques).

Now let $p_1,p_2, \ldots$ be the points on one ray sorted by decreasing distance to the origin, and let $q_1,q_2, \ldots$ be the points on a different ray, again sorted by decreasing distance to the origin. If we connect $p_i$ with $q_j$ then we have points $p_1,p_2, \ldots , p_{i-1},q_1,q_2, \ldots ,q_{j-1}$ on one side and all points of the third remaining ray on the other side. That is, the pair $\{p_i,q_j\}$ is *k*-deep, provided $i+j-2 \ge k$. This holds if $i \ge \lceil \frac{k}{2} \rceil + 1$ and $j \ge \lfloor \frac{k}{2} \rfloor + 1$, since $\lceil \frac{k}{2} \rceil + 1 +\lfloor \frac{k}{2} \rfloor + 1 = k+2$.

This shows that if we remove the $\lceil \frac{k}{2} \rceil$ furthest points on the first ray, the $\lceil \frac{k}{2} \rceil$ furthest points on a second ray, and the $\lfloor \frac{k}{2} \rfloor$ furthest points on the remaining third ray, then we have a clique in $\textsf{G}_{k}\hspace{-0.05em}(P)$ of size $n - 2\lceil \frac{k}{2} \rceil -\lfloor \frac{k}{2} \rfloor = n-\lceil \frac{3k}{2} \rceil$. $\square$

It is worthwhile to mention that these tripod point sets showing tightness are the same sets that show that the lower bound of  on the number of $(\le k)$-edges is tight for $k\le \frac{n}{3}$ (see [1, 2, 5, 12]).

## Halving Cliques—Proof of Theorem 1.3

We move to the extreme case of $\left( \frac{n}{2}-1\right)$-deep cliques, for *n* even, i.e., these are cliques of pairs carrying lines perfectly halving the point set. It appears to be advantageous to flip perspective: Rather than asking for the maximal size of a $\left( \frac{n}{2}-1\right)$-deep clique in a set of *n* points, we ask for the minimal number of points one has to add to an *m*-point set, so that it becomes such a clique of the extended set.

### Definition 4.1

(*halving pair, edge, and clique*)

- Given a point set *P* in general position, an unordered pair  is called a *halving pair* (of *P*), if the line through *p* and *q* has the same number of points of *P* on either side (which can happen for *n* even only). An ordered pair *pq* is called a *halving edge* (of *P*), if $\{p,q\}$ is a halving pair.
- Given a point set *P* in general position, a subset *Q* is called a *halving clique*, if all pairs in  are halving pairs of *P* (equivalently, if *Q* is a $\left( \frac{n}{2}-1\right)$-deep clique).
- Given a set *Q* of *m* points in general position, we define $\gamma (Q)$ as the smallest size of a superset *P* with *Q* a halving clique of *P*.
- For $m \in \mathbb {N}$, we let $\gamma (m)$ be the minimum of $\gamma (Q)$ over all sets *Q* of *m* points in general position, and we let $\gamma ^\textsf{c}(m)$ be the minimum of $\gamma (Q)$ over all sets *Q* of size *m* in convex position.

**Fig. 5:**
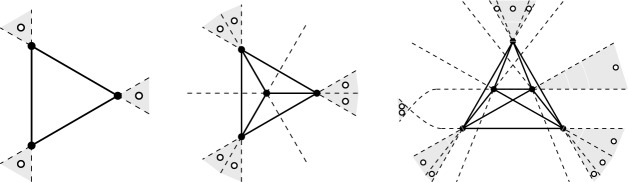
Sets of 3, 4, and 5 points, resp., extended to a set which makes them halving cliques, showing $\gamma (3) \le 6$, $\gamma (4) \le 10$, and $\gamma (5) \le 16$. We will see (Lemma 4.3) that these are tight examples

Observe that $\gamma (Q)$ need not be defined, e.g., if *Q* has two lines connecting pairs of points in *Q* which are parallel (we will prove in Lemma 4.7 that this is actually the only reason for $\gamma (Q)$ not to be defined). If defined, $\gamma (Q)$ always has to be even. Figure 5 gives a few examples of deep halving cliques in point sets. Note that the 5-point set in Fig. 5 rightmost has, as drawn, two parallel connecting lines, but it is obvious that we can perturb the points such that one obtains the type of arrangement of connecting lines as shown.

### Lower bounds on $\gamma (m)$ and $\gamma ^\textsf{c}(m)$

A lower bound of $\lfloor \frac{m^2}{2}\rfloor$ on $\gamma (m)$ is already mentioned in [9] (the short argument given in the first paragraph of the proof of Lemma 4.3 might be considered to be more concise). For our slight improvement to $\left\lfloor \frac{(m-1)(m+3)}{2}\right\rfloor$ we need another basic lemma on *j*-edges, as it can be found already in [6, 11] (see, e.g., [7, Lem. 4.3]). Note that these quadratic bounds are better than the bound of $\gamma (Q) = \Omega (m^{3/2})$ as it follows from Dey’s upper bound of $O(n^{4/3})$ on the number of halving pairs.

#### Lemma 4.2

(Lovász Lemma for j-edges) Let *h* be a directed line disjoint from *P* which separates *P* into a set *L* left of *h* and a set *R* right of *h*. For $0 \le j \le \frac{n}{2}-1$, the number of *j*-edges *pq* that intersect *h* from left to right (i.e., $p \in L$ and $q \in R$) is exactly $\min \{j+1, |L|, |R|\}$.

#### Lemma 4.3

$$\gamma (m) \ge \left\lfloor \frac{(m-1)(m+3)}{2}\right\rfloor \ge \left\lfloor \frac{m^2}{2}\right\rfloor $$.

#### Proof

Let *Q* be a halving clique in *P*, with $|Q| = m$ and $n:= |P|$. On the one hand, by Lemma 4.2, any line can separate at most $\frac{n}{2}$ halving pairs in *P*. On the other hand, if *Q* is split by a line $\ell$ into sets of size $\lfloor \frac{m}{2} \rfloor$ and $\lceil \frac{m}{2} \rceil$, resp., then $\ell$ separates at least $\lfloor \frac{m}{2} \rfloor \cdot \lceil \frac{m}{2} \rceil$ halving pairs of *P*. Hence $\lfloor \frac{m}{2} \rfloor \cdot \lceil \frac{m}{2} \rceil \le \frac{n}{2}$ and a bound of $\gamma (m) \ge \lfloor \frac{m^2}{2}\rfloor$ readily follows.^4^

For the slightly stronger bound claimed in the lemma, the line $\ell$ has to be chosen more carefully. First consider a point $q \in Q$ that is extreme in *Q*. This point has $m-1$ halving pairs (with respect to *P*) with the other points in *Q*. Choose a line *t* tangent to the convex hull of *Q* with $P \cap t = \{q\}$. This line has at least $m-1$ halving pairs with points in its halfplane containing *Q*, and it follows that it must have at least $m-2$ halving pairs with points in its other halfplane (see Lemma 2.3). We choose $\ell$ close to *q* in such a way that (i) it divides *Q* into sets of size $\lfloor \frac{m}{2} \rfloor$ and $\lceil \frac{m}{2} \rceil$, resp., and it splits at least half, i.e., at least $\lceil \frac{m-2}{2} \rceil$ of the extra halving pairs incident to *q* in the other halfplane of *t*. This line $\ell$ splits now at least $\lfloor \frac{m}{2} \rfloor \cdot \lceil \frac{m}{2} \rceil + \lceil \frac{m-2}{2} \rceil$ halving pairs of *P*. That is,

$$\begin{aligned} \left\lfloor \frac{m}{2} \right\rfloor \cdot \bigg \lceil \frac{m}{2} \bigg \rceil + \bigg \lceil \frac{m-2}{2} \bigg \rceil \le \frac{n}{2} ~~~\Leftrightarrow ~~~ \left\lfloor \frac{(m-1)(m+3)}{2} \right\rfloor \le n \end{aligned}$$

as can be readily shown.^5^$\square$

This yields $\gamma (3) \ge 6$, $\gamma (4) \ge 10$, and $\gamma (5) \ge 16$, which are tight by the examples given in Fig. 5. We do not expect the bound to be tight in general. For the next value the bound in the lemma reads $\gamma (6) \ge 22$ while the best construction we can give is a 6-point set *Q* for with $\gamma (Q) = 24$, see Fig. 8.

#### Lemma 4.4

For $m \in \mathbb {N}$, $\gamma ^\textsf{c}(m) \ge m(m-1)$, i.e., if a set *Q* of *m* points in convex position is a halving clique in a set *P* in general position, then $|P| \ge m(m-1)$.

#### Proof

The basic method we use here is a refinement of what is called chain-method by Khovanova and Yang in [9], and which goes back to Tamal Dey’s paper [4] with upper bounds on the number of *j*-edges (there called *k*-*set edges*). While the chains in [4, 9] are *x*-monotone and convex, our chains continue further and they are actually closed curves, potentially self-intersecting. This refinement is essential for obtaining the tight bounds we obtain in this lemma.

**Fig. 6:**
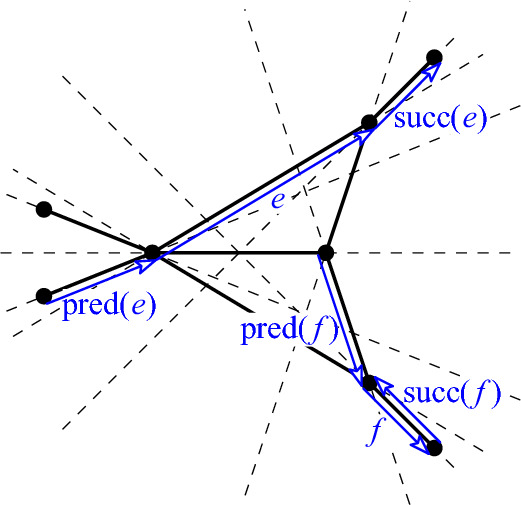
A graph of halving pairs for a set of 10 points, and halving edges *e* and *f* with their respective successors and predecessors. Note that in this example, the sequence $\sigma (e)$ runs through all halving edges before it returns to *e*

Consider a halving edge $e=pq$ (an ordered pair) of *P*. Let *h* be a directed line through *p* and *q* directed from *p* to *q*. Start rotating *h* about *q* in counterclockwise direction. Let $q'$ be the first point hit such that $e'=qq'$ is a halving edge; note that as this happens, $q'$ has to follow *q* in the direction of the rotating line. (This follows from the Alternation Lemma, see [7, Lem. 4.1]: As we rotate a directed line about a point *q*, halving edges are met in alternation after and before *q* (in the given direction of the line).) Call this edge $e'$ the *successor*, $\textsf{succ}(e)$, of *e* (see Fig. 6). Similarly, rotate *h* in clockwise direction, now about *p*, until the first point $p'$ is met which forms a halving edge $e''=p'p$. Call this edge $e''$ the *predecessor*, $\textsf{pred}(e)$, of *e*. Observe that $\textsf{pred}(\textsf{succ}(e))= e$. Note that $\textsf{succ}(pq)$ can potentially be *qp* (see, e.g., edge *f* in Fig. 6). In fact, this will happen if and only if *q* is involved in a single halving pair (hence two halving edges), as it is the case for all extreme points of *P*. Let us emphasize, that these relations are on *directed* edges, *not unordered* pairs.

In this way, every halving edge *e* generates an infinite sequence $\sigma (e):= (e_0:=e,e_1,e_2, \ldots )$ of halving edges with $e_{i+1} = \textsf{succ}(e_i)$, $i \in \mathbb {N}_0$. This sequence has to eventually return to *e*: To see this, let *j* be the first index $j \in \mathbb {N}$ with $e_j = e_i$ for $i<j$. If $i=0$, the claim is established. Otherwise, we have $\textsf{pred}(e_j) = \textsf{pred}(e_i)$, and hence $e_{j-1} = e_{i-1}$ (recall $\textsf{pred}(\textsf{succ}(e))= e$), in contradiction to our choice of *j*.

The proof aims at injectively assigning to every halving edge $e=pq$ with $p,q \in Q$ a point $\chi (e)$ in *P*. Since there are $m(m-1)$ halving edges formed by *Q*, this will show $|P| \ge m(m-1)$ and the bound follows. The point $\chi (e)$ will be found by following the sequence $\sigma (e)$ up to a certain carefully chosen point and then choose $\chi (e)$ as this last point reached. In order to specify this “certain point”, we need another relation.

We define a partial order on the points in *P* not in the interior of *conv*(*Q*) as follows: we say that $p'$ *dominates* *p*, $p \preceq p'$, if $p'=p$ or *p* is in the interior of the convex hull of $Q \cup \{p'\}$. Clearly, this is a partial order, with all elements in *Q* minimal (but there can be other minimal elements), and all extreme points of *P* maximal (but there can be other maximal elements).

Now, $\chi (e)$ is chosen as follows. We proceed through $\sigma (e)$ until we get for the first time to an edge $q'q''$ such that $q' \not \preceq q''$. Then $\chi (e):=q'$.

- $\chi$ *is well-defined.* The sequence $\sigma (e)$ of successive successors starting from $e=pq$ will eventually return to *e*. For $p'p:= \textsf{pred}(e)$, note that $p' \not \preceq p$, since $p \in Q$ and we agreed that the elements in *Q* are minimal elements of our dominance order.
- $\chi$ *is injective.* Consider a point *q* and consider the incoming halving edges in the convex hull of $Q \cup \{q\}$. The two tangents to *conv*(*Q*) through *q* define four wedges (cones). Among those, there is the wegde $W_{in}$ containing *conv*(*Q*), and its opposite wedge $W_{out}$. As a line through *q* lies in the union of these two wedges and rotates, it encounters halving edges alternatingly in $W_{in}$ and in $W_{out}$: If, while rotating in counterclockwise direction, it meets an incoming edge *e* in $W_{in}$, it meets next an outgoing edge, namely $\textsf{succ}(e)$, in $W_{out}$, then an incoming edge in $W_{in}$, and so on; see the Alternation Lemma as mentioned and used in the second paragraph of this proof. Only the last incoming edge from $W_{in}$ might have its successor not in $W_{out}$. It follows easily that a path in search for $\chi (e)$ of an edge in $Q^2$ can stop in *q* only if the path comes to *p* along this last edge incoming to *p* within $W_{in}$. And therefore, $|\chi ^{-1}(q)|\le 1$.$\square$

**Fig. 7:**
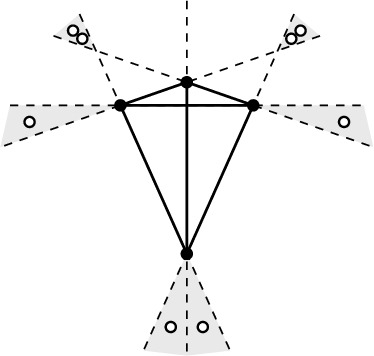
A set *Q* of 4 points in convex position with $\gamma (Q) = 12 = \gamma ^\textsf{c}(4)$

We see that the example in Fig. 7 has minimal size $12=4\cdot 3$ for a set with a halving clique of size 4 in convex position, which is therefore a tight example for $m=4$ in Lemma 4.4. We compare this to the halving clique of size 4 in 10 points Fig. 5 middle, which shows that nonconvex halving cliques can appear in smaller sets.

### Lower Bound Arguments for $\gamma (Q)$

**Fig. 8:**
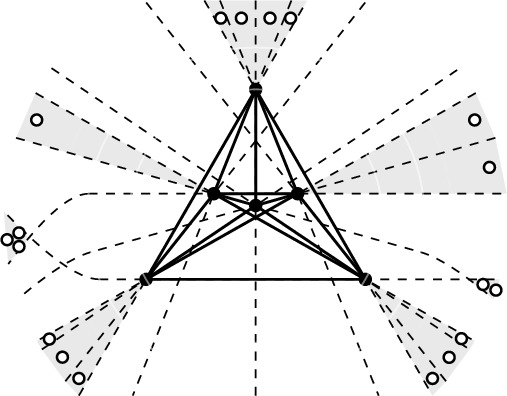
A halving clique *Q* of size 6 in a set of 24 points

In preparation of showing that the bound in Lemma 4.4 is tight, we consider the example in Fig. 8: A set *Q* of 6 points with triangular convex hull, embedded in a set of 24 points where *Q* is a halving clique. We want to argue that at least 24 points are needed for this set *Q* to be a halving clique, i.e., $\gamma (Q) = 24$. We will also apply the arguments to a set *Q* of 5 points in convex position with $\gamma (Q) = 24$, compared to the $4\cdot 5 = 20$ bound given in Lemma 4.4. The punchline is that, still, the same lower bound arguments will help in showing the existence of sets in convex position which realize the lower bound from Lemma 4.4.

#### Definition 4.5

(*avoiding, backyard, front yard*) Let $\pi =\{p,q\}$ and $\pi '=\{p',q'\}$ be pairs of two distinct points each in the plane, with $\pi \ne \pi '$.

**Fig. 9:**
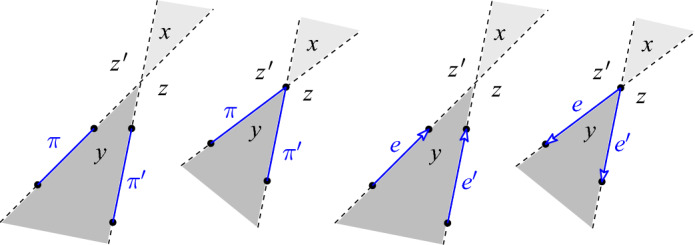
Backyard (shaded) and front yard (darker shaded) of pairs of avoiding unordered pairs ($\pi$ and $\pi '$) or avoiding ordered edges (*e* and $e'$). *x*, *y*, *z*, and $z'$ denote the number of points in the respective open wedges

- We call $\pi$ and $\pi '$ *avoiding*, (i) if they share exactly one point, or (ii) if they are disjoint and the line through *p* and *q* avoids the segment spanned by $p'$ and $q'$, and vice versa.
- Let $\pi$ and $\pi '$ be avoiding, and let $\ell$ and $\ell '$ be the lines carrying $\{p,q\}$ and $\{p',q'\}$, resp. The complement of these two lines in the plane decomposes into four open wedges (or three slabs, if $\ell$ and $\ell '$ are parallel). The wedge (or slab) with $\{p,q\} \cup \{p',q'\}$ on its boundary is called the *front yard* of $\pi$ and $\pi '$. Unless $\ell$ and $\ell '$ are parallel, the wedge antipodal to the front yard is called the *backyard *of $\pi$ and $\pi '$. See Fig. 9 for an illustration.

Let *pq* and $p'q'$ be two ordered edges of points, with $\{p,q\} \ne \{p',q'\}$.

- We call *pq* and $p'q'$ *avoiding*, if the underlying unordered pairs $\{p,q\}$ and $\{p',q'\}$ are avoiding, and *pq* and $p'q'$ are either both directed towards the intersection of the lines carrying *pq* and $p'q'$, or they are both directed away from this intersection.
- For *pq* and $p'q'$ avoiding, its *front yard* and *backyard* are the same as front yard and backyard, resp., of the underlying unordered pairs $\{p,q\}$ and $\{p',q'\}$.

Observe that if *Q* is in convex position, then any two distinct pairs of points in ${Q \atopwithdelims ()2}$ are avoiding. Here is a simple observation about the yards of pairs of avoiding halving pairs or edges in a point set, see Fig. 9.

#### Observation 4.6

Let *P* be a finite set of points in general position in the plane.

(i) Let $e, e'$ be two avoiding directed edges of points in *P*, with *x* the number of points in *P* in the backyard and *y* the number of points in *P* in the front yard. Suppose *e* is a *k*-edge. If *e* and $e'$ are disjoint, then $e'$ is a *k*-edge iff $x=y+2$, and if they share a point, then $e'$ is a *k*-edge iff $x=y+1$.
(ii) Let $\pi , \pi ' \in {P \atopwithdelims ()2}$ be two avoiding pairs, with *x* the number of points in *P* in the backyard and *y* the number of points in *P* in the front yard. Suppose $\pi$ is a halving edge. If $\pi$ and $\pi '$ are disjoint, then $\pi '$ is a halving pair iff $x=y+2$, and if they share a point, then $\pi '$ is a halving pair iff $x=y+1$.

#### Proof

See Fig. 9 and consider the situation of *e* and $e'$ not sharing an endpoint as in Fig. 9  (third from left). If *e* is a *k*-edge, then $x+z'=k$. $e'$ is a $(y+z'+2)$-edge and therefore this is a *k*-edge as well iff $x=y+2$. A similar argument works for *e* and $e'$ sharing an endpoint. Also the claim for the unordered pairs follows easily. $\square$

Let *Q* be a halving clique in a set *P* and let $\pi$ and $\pi '$ be avoiding pairs in *Q*, with *j* points from *Q* in its front yard. The observation says, in particular, that the backyard of $\pi$ and $\pi '$ must contain at least $j+2$ points, or at least $j+1$ points in *P*, depending in whether the two pairs are disjoint or not. Note now that the shaded areas in Fig. 8 are backyards of pairs of halving pairs, these backyards are pairwise disjoint, and they contain the minimal number of points enforced by *Q* by Observation 4.6. It follows that the set of 24 points is of minimal size for a set with the given set *Q* as halving clique and $\gamma (Q) = 24$.

**Fig. 10:**
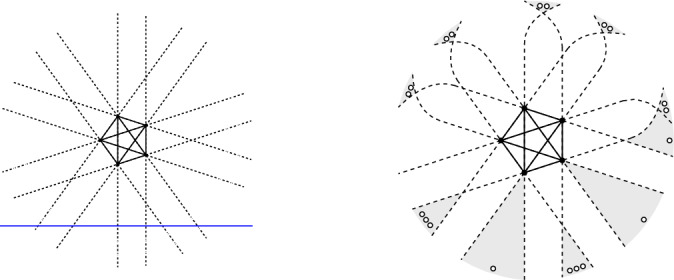
A set *Q* of 5 points in convex position, obtained by a projective transformation from a regular pentagon. The set is embedded in 24 points where it forms a halving clique. Less than 24 are not possible for this specific set *Q* in convex position, i.e., $\gamma (Q) = 24$

Let us apply this lower bound argument to a specific set of 5 points in convex position, see Fig. 10. We start with the vertex set *Q* of a regular pentagon. *Q* is in general position, but it has parallel connecting lines. Therefore, *Q* cannot possibly be extended to a set $P \supseteq Q$ where *Q* is a halving clique. Hence, we consider a controlled perturbation $\tilde{Q}$ of *Q*: We choose a line (not parallel to any connecting line) which has *Q* and all intersections of its connecting lines on one side, see Fig. 10 left, and apply a projective transformation that sends this line to infinity. (This idea of a controlled perturbation by projective transformation can be found already in [9]). In this way we get the arrangement of connecting lines as indicated schematically in Fig. 10 right. The points added, 19 of them, are arranged in 9 disjoint backyards^6^ of pairs of pairs of points in $\tilde{Q}$, where in each backyard the number is the minimum required by $\tilde{Q}$. Therefore, if the perturbed set $\tilde{Q}$ is extended to a set where $\tilde{Q}$ is a halving clique, this has to contain at least 24 points, which exceeds the lower bound of $5 \cdot 4 = 20$ in Lemma 4.4.

**Fig. 11:**
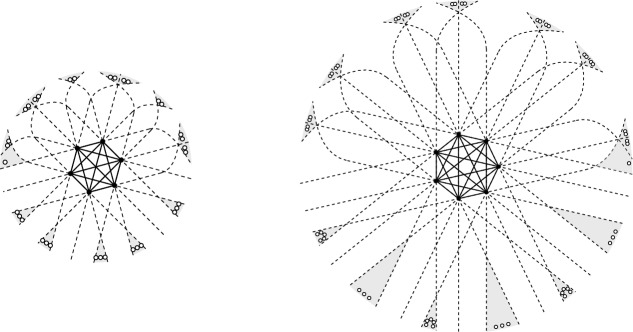
Projective perturbations $\tilde{Q}_m$ of regular *m*-gon with $\gamma (\tilde{Q}_m) = 2(m-1)(m-2)$

The analysis of this construction via projective transformation of a regular *m*-gon can be generalized, see Fig. 11, and yields sets $\tilde{Q}_m$ of *m* points in convex position with $\gamma (\tilde{Q}_m)=2(m-1)(m-2)$ (see also the construction of an extension of the same set in [9, Thm. 5.9] yielding a set with $2m^2-5m$ points, for *m* even). This, however, does not contradict the tightness of Lemma 4.4. Rather, as we will see, the value of $\gamma (Q)$ is not determined by the order type of *Q*. Instead $\gamma (Q)$ depends on the arrangement of connecting lines. There are different carefully chosen sets $Q_m$ of *m* points in convex position with $\gamma (Q_m)=m(m-1)$.

### Extending Sets

We will show next how to extend any set *Q* in general position without parallel connecting lines to a small set *P* where *Q* is a halving clique. A bound of $m(m^2-2m+2)$ on the size of *P* was shown in [9, Thm. 5.8], which we improve by a factor of 2. More importantly, the idea of the proof will be used for the tight bound in Lemma 4.8 below.

#### Lemma 4.7

For every set *Q* of *m* points in general position without parallel connecting lines we have $\gamma (Q) \le \frac{m}{2}(m^2-3m+4)$.

#### Proof

The $$m(m-1)$$ directed edges spanned by *Q* have a cyclic ordering $$(e_0,e_1,\ldots , $$$$ e_{m(m-1)-1})$$ sorted by angle in counter clockwise direction, say. For all $$0 \le i \le {m \atopwithdelims ()2} - 1$$, edges $$e_i$$ and $$e_{i + {m \atopwithdelims ()2}}$$ have the same underlying unordered pair, directed in opposite directions.

If we can ensure that after extending *Q* to a set $P \supseteq Q$

1 $$\begin{aligned} \text{ for } \text{ all } i=1,2,\ldots ,{m\atopwithdelims ()2}, e_{i-1} \text{ and } e_i \text{ are } k_i-\text{ edges } \text{ for } \text{ some } \text{ but } \text{ the } \text{ same } k_i, \end{aligned}$$

then clearly all edges $e_0,e_1,\ldots , e_{m\atopwithdelims ()2}$ are *k*-edges for the same *k*. Note that in particular, $e_0$ and $e_{m\atopwithdelims ()2}$ are *k*-edges for the same *k*, while they are opposite orientations of the same underlying pair. This is possible only if both $e_0$ and $e_{m\atopwithdelims ()2}$ are halving. Hence the goal is set: In order to extend *Q* to a set *P* where *Q* is a halving clique, all we need is to guarantee Property (1).

It is easily seen that any two consecutive edges in the cyclic ordering form an avoiding pair. This can be seen by simply observing this for sets of 4 points, from which the general claim readily follows. Moreover, the backyard of each such consecutive pair is empty, and the front yard is empty, provided the two edges share an endpoint.

Following Observation 4.6, we place for each pair $e_{i-1}$ and $e_i$ the right number of points in the backyard, so that they are $k_i$-edges for the same $k_i$. In order to avoid interference of these additions, we observe that the lines carrying $e_{i-1}$ and $e_i$, resp., bound a common infinite cell of the arrangement of connecting lines of *Q*, a consequence of our ordering by angle. If we place the required points in the infinite cell contained in the backyard, these points cannot end up in the front yard of some other pair. This concludes the construction.

Finally, we observe that for each of the ${m \atopwithdelims ()2}$ pairs of edges considered, we add at most $m-2$ points, therefore at most ${m \atopwithdelims ()2}(m-2)$ points altogether are added for the construction of *P*, and thus $P \le m + {m \atopwithdelims ()2}(m-2) = \frac{m}{2}(m^2-3\,m+4)$. $\square$

The recipe described in the proof of Lemma 4.7 was employed to generate the examples in Figs. 5, 8, and 10. We are now ready to prove tightness of Lemma 4.4.

#### Lemma 4.8

For every $m \in \mathbb {N}$, $m \ge 2$, there is a set $Q_m$ of *m* points in convex position with $\gamma (Q_m) = m(m-1)$. That is, there is a set $P_m \supseteq Q_m$ of $m(m-1)$ points which contains $Q_m$ as halving clique.

#### Proof

For the construction of $Q_m$, let *C* be the curve $\{(x,x^2)\in \mathbb {R}^2 \mid x \ge 0\}$; its essential properties are that it is an *x*- and *y*-monotone strictly convex curve of increasing slope. We choose an *x*-monotone sequence of *m* points $(p_1, p_2, \ldots , p_m)$ on *C*, with the extra condition that

2 $$\begin{aligned} \text{ for } \text{ all } j \ge 3\text{, } \text{ the } \text{ slope } \text{ of } p_1p_j \text{ is } \text{ larger } \text{ than } \text{ the } \text{ slope } \text{ of } p_{j-2}p_{j-1}\text{. } \end{aligned}$$

**Fig. 12:**
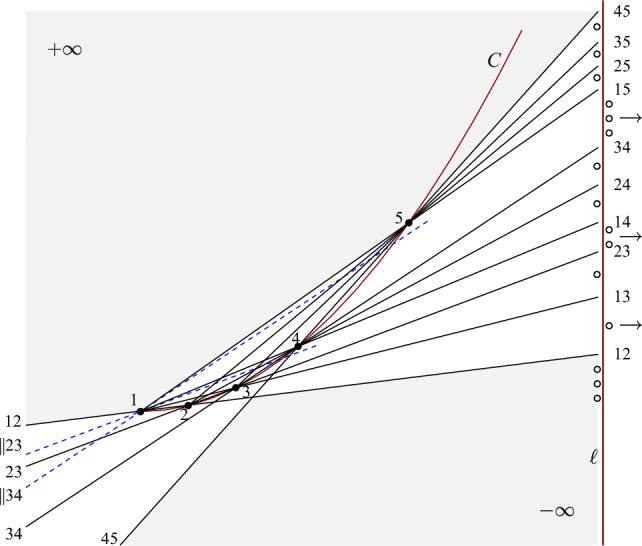
Sets $Q_m$ of *m* points in convex position with $\gamma (Q_m)=m(m-1)$ (here $m=5$). For the points extending $Q_m$ displayed to the right in infinite cells, the points right of the vertical line should be placed in the corresponding antipodal infinite cell of the arrangement of connecting lines

This is easy to attain by simply letting $p_j$ lie strictly above the line through $p_1$ parallel to $p_{j-2}p_{j-1}$ (see Fig. 12). (A direct way of attaining the conditions is to set $p_1 = (0,0)$ and for $i>1$, $p_i = (2^{i-1},2^{2(i-1)})$.) From now on let us write *i* short for point $p_i$ in $Q_m$.

The ${m \atopwithdelims ()2}$ directed edges *ij*, $1 \le i < j \le m$, sorted by slope, give the sequence

$$\begin{aligned} \underbrace{{\,\,12\,\,}}_{\textrm{block}~2} \underbrace{{\,\,13\,\,} {\,\,23\,\,}}_{\textrm{block}~3} \underbrace{{\,\,14\,\,} {\,\,24\,\,} {\,\,34\,\,}}_{\textrm{block}~4} \underbrace{ {\,\,15\,\,} {\,\,25\,\,} {\,\,35\,\,} {\,\,45\,\,}}_{\textrm{block}~5} \ldots \underbrace{ {\,\,1m\,\,} {\,\,2m\,\,} \cdots {\,\,(m-2)m\,\,} {\,\,(m-1)m\,\,}}_{\textrm{block}~m}, \end{aligned}$$

where we arranged the sequence in blocks according to the larger index occurring in a pair (hence there is no block 1): The order in each block is given, since *C* is a convex curve of increasing slope. The extra relations of 23 vs. 14, 34 vs. 15, ..., $(j-2)(j-1)$ vs. 1*j*, are enforced by condition (2) above.

This is the order in which the  lines connecting pairs of points in $Q_m$ meet a vertical line $\ell$ right of all crossings of pairwise connecting lines. Similar to the proof of Lemma 4.7, we are left to place points in the infinite cells of the pairwise connecting lines arrangement so that *Q* becomes a halving clique, see Fig. 12 and (3).

3 $$\begin{aligned} ^{(m-1)m} \, \underbrace{\circ \circ \cdots \circ }_{m-2} \, \underbrace{^{12}}_{0} \, \overbrace{\circ }^{1}\underbrace{ ^{13} \circ ^{23}}_{1} \, \overbrace{\circ \circ }^{2} \, \underbrace{^{14} \circ ^{24}\circ ^{34}}_{2} \ldots \, \overbrace{\circ \circ \cdots \circ }^{j-2} \, \underbrace{ ^{1j} \circ ^{2j}\circ \cdots ^{(j-2)j} \circ ^{(j-1)j}}_{j-2} \ldots \end{aligned}$$

(i) The lowest cell of the arrangement is the backyard of the avoiding pair  and therefore requires $m-2$ points (all points in  lie in the front yard of this pair).
(ii) Within block *j*, $3 \le j \le m$, the line $\ell$ intersects the backyard of the avoiding pair $\{ij, (i+1)j\}$, $1 \le i \le j-2$, with its front yard empty. We place one point in the corresponding infinite backyard cell of the arrangement, this gives $j-2$ points altogether for block *j*.
(iii) For a pair $\{(j-2)(j-1),1j\}$, $3 \le j \le m$, between blocks $j-1$ and *j*, $\ell$ intersects the front yard of this avoiding pair with points $2,3,\ldots ,j-3$, i.e., $j-4$ of them. Therefore we have to place $(j-4) + 2 = j-2$ points in the infinite cell in its backyard, which is the cell antipodal to the infinite cell intersected by $\ell$. For these pairs, in Fig. 12 the required points are still drawn next to $\ell$, but positioned right of $\ell$ with an arrow indicating that they should actually go to the antipodal cell.

Summing up, this givespoints in the set extending *Q*, where *Q* is a halving clique. $\square$

## Bounds for *k* Close to $\frac{n}{2}$—Proof of Theorem 1.2(iii)

We conclude by addressing the case of *k* close to $\frac{n}{2}$.

### Lemma 5.1

(upper bound in Theorem 1.2(iii)) For $n \in \mathbb {N}$ and $k \in \mathbb {N}_0$, $k \le \lfloor \frac{n}{2} \rfloor -1$, a *k*-deep clique in any set *P* of *n* points has size at most $2 \sqrt{n(\lfloor \frac{n}{2} \rfloor - k)}$.

### Proof

We can assume that *P* is in general position. On the one hand, by Lemma 4.2, any line can separate at most

$$\begin{aligned}  &   \left\{ \begin{array}{ll} 2\left( (k+1)+ \ldots +(\frac{n}{2}-1)\right) + \frac{n}{2}&  \text{ for } \text{ n } \text{ even }\\ 2\left( (k+1)+ \ldots +\lfloor \frac{n}{2}\rfloor \right) &  \text{ for } \text{ n } \text{ odd } \end{array} \right\} \\= &   \lfloor \frac{n}{2} \rfloor \cdot \lceil \frac{n}{2} \rceil - k(k+1) \le \left( \lceil \frac{n}{2} \rceil + k\right) \left( \lfloor \frac{n}{2} \rfloor - k\right) \\\le &   (n-1)\left( \lfloor \frac{n}{2} \rfloor -k\right) \le n\left( \lfloor \frac{n}{2} \rfloor -k\right) - 1 \end{aligned}$$

*k*-deep pairs of *P*. On the other hand, if a *k*-deep clique of size *m* is split by a line $\ell$ into sets of size $\lfloor \frac{m}{2} \rfloor$ and $\lceil \frac{m}{2} \rceil$, resp., then $\ell$ separates at least $\lfloor \frac{m}{2} \rfloor \cdot \lceil \frac{m}{2} \rceil$ *k*-deep pairs of *P*. Hence, $\lfloor \frac{m}{2} \rfloor \cdot \lceil \frac{m}{2} \rceil \le n\left( \lfloor \frac{n}{2} \rfloor -k\right) -1$ and the lemma readily follows (note $\frac{m^2-1}{4} \le \lfloor \frac{m}{2} \rfloor \cdot \lceil \frac{m}{2} \rceil$). $\square$

We are left to show that this bound is asymptotically tight. First, we show how to blow up examples of large halving cliques.

### Lemma 5.2

For every $m,c \in \mathbb {N}$ and $x \in \mathbb {N}_0$, there is a set $Q_{m,c}$ of *mc* points and a superset $P_{m,c,x} \supseteq Q_{m,c}$ of $n:=cm(m-1)+2x$ points for which $Q_{m,c}$ is a *k*-deep clique in $P_{m,c,x}$ with $k = \frac{n}{2}-c = c(\frac{m(m-1)}{2}-1)+x$.

### Proof

Using Lemma 4.8 start with sets $P_m,Q_m$ such that $P_m$ has $m(m-1)$ points and $Q_m$ is a $(\frac{m(m-1)}{2}-1)$-deep clique of $P_m$ with *m* points. Assume $P_m$ is in general position (perturbing it if necessary). For each point $p\in P_m$, define a line $\ell _p$ passing through *p* that keeps at least $|P_m|/2 - 1$ points of $P_m$ on each side. Construct $Q_{m,c}$ (of size *cm*) and $P_{m,c}$ (of size $cm(m-1)$) by replacing each point *p* by a cluster of *c* points on line $\ell _p$, sufficiently close to *p*. To be more precise, we require that if we connect $p'$ in the *p*-cluster with $q'$ in the *q*-cluster, then the line through $p'$ and $q'$ maintains the same relative position to other clusters as the line through *p* and *q*. In particular any pair taken from distinct clusters in $Q_{m,c}$ will keep at least $|P_m|/2 - 1$ clusters, and thus $c(|P_m|/2 - 1) = c(\frac{m(m-1)}{2} - 1)$ points on each side. Furthermore, the same will be true for any pair of points drawn from the same cluster. Thus the set $Q_{m,c}$ forms a clique of depth $c(\frac{m(m-1)}{2} - 1)$.

Now, in order to extend $P_{m,c}$ to $P_{m,c,x}$ of size $n = cm(m-1) +2x$, we choose two antipodal infinite cells in the arrangement of lines connecting pairs of points in $Q_{m,c}$. By placing *x* points in each of these two cells, every pair in $Q_{m,c}$ increases its depth by *x*, and thus $Q_{m,c}$ is a *k*-deep clique with $k = c(\frac{m(m-1)}{2} - 1) + x = n/2 - c$. $\square$

### Lemma 5.3

For all $n \in \mathbb {N}$ and $k \in \mathbb {N}_0$ with $k \le \lfloor \frac{n}{2} \rfloor -1$, there exists a set of *n* points with a *k*-deep clique of size $\Omega \left( \sqrt{n(\lfloor \frac{n}{2} \rfloor -k)}\,\right)$.

### Proof

We assume $k \ge \frac{n}{3}$, since otherwise the claim is obvious by Theorem 1.2(ii).

For a parameter $m\ge 3$ to be determined later, set $M= M(m):= \frac{m(m-1)}{2}$ and choose $c = c(m):= \lfloor \frac{k}{M-1} \rfloor = \frac{k-x}{M-1}$ with $0 \le x < M-1$. Assume $c\ge 1$. If we choose $Q_{m,c}$ (of size *cm*) and $P_{m,c,x}$ (of size $2cM+2x$) as in Lemma 5.2 above, then $Q_{m,c}$ is a $(c(M -1)+x)$-deep clique, where $c(M -1) +x = k$. We want to make sure that $P_{m,c,x}$ does not end up too large, i.e., $cM + x \le \frac{n}{2}$. We show that this is attained for $m = \bigg \lceil \sqrt{\frac{n}{\lfloor \frac{n}{2}\rfloor - k}} \bigg \rceil + 1$.

$$\begin{aligned}  &   m = \bigg \lceil \sqrt{\frac{n}{\lfloor \frac{n}{2}\rfloor - k}} \bigg \rceil + 1 ~~ \Rightarrow ~~ \underbrace{m(m-1)}_{2M} \ge \frac{n}{\lfloor \frac{n}{2}\rfloor - k}\\\Rightarrow &   M \ge \frac{\frac{n}{2}}{\frac{n}{2} - k} ~~ \Leftrightarrow ~~ k \frac{M}{M-1} \le \frac{n}{2}\\\Rightarrow &   k \frac{M}{M-1} + x \underbrace{\left( 1 - \frac{M}{M-1}\right) }_{< 0} \le \frac{n}{2} ~~\Leftrightarrow ~~ \frac{k-x}{M-1} \cdot M + x \le \frac{n}{2}. \end{aligned}$$

It remains to analyze *cm*, the size of $Q_{m,c}$. With the assumption of $k \ge \frac{n}{3}$, we get

$$\begin{aligned} cm = \left\lfloor \frac{k}{M-1} \right\rfloor m = \Omega \left( \frac{km}{M-1}\right) = \Omega \left( \frac{k}{m}\right) = \Omega \left( \frac{n}{m}\right) = \Omega \left( \sqrt{n (\lfloor \frac{n}{2}\rfloor - k)}\right) . \end{aligned}$$

If $|P_{m,c,x}| < n$, we can add points arbitrarily to extend it to a set of size *n*. $\square$

## Footnotes

1 The Tukey depth [15] of a point *q* relative to a point set *P* can be defined as the minimum number of points of *P* in any closed halfspace containing *q*, or equivalently, with *q* on its boundary. The latter definition, which forces *q* on the boundary, generalizes to depth of a pair $\{p,q\}$ considered here – now we force both *p* and *q* on the boundary –, except that we consider open halfspaces.

2 At the ComPoSe “Workshop on the Erdős-Szekeres Theorem: Extensions and Variations”, at the International Centre for Mathematical meetings (CIEM), Castro Urdiales, Spain, May 23rd to 27th, 2011.

3 The theorem in [3, Thm. 1] is formulated for *k*-sets rather than *k*-edges. A *k*-set is a subset of *P* that can be separated from its complement in *P* by a line disjoint from *P*. It is known that the number of *k*-sets equals the number of $(k-1)$-edges. The bound of *kn* on the number of $(\le k)$-sets in [3] for $k < \frac{n}{2}$ therefore translates to a bound of *kn* on the number of $(k-1)$-edges for $k < \frac{n}{2}$.

4 Note that $2\cdot \lfloor \frac{m}{2} \rfloor \cdot \lceil \frac{m}{2} \rceil$ equals $\frac{m^2}{2} = \left\lfloor \frac{m^2}{2}\right\rfloor$ for *m* even and it equals $\frac{m^2-1}{2} =\left\lfloor \frac{m^2}{2}\right\rfloor$ for *m* odd.

5 $2(\lfloor \frac{m}{2} \rfloor \cdot \lceil \frac{m}{2} \rceil + \lceil \frac{m-2}{2} \rceil )$ equals $\frac{(m-1)(m+3)}{2}$ for *m* odd, and it equals $\frac{(m-1)(m+3)}{2} - \frac{1}{2}$ for *m* even.

6 Note that one of the backyards with 3 points actually contains another backyard with 2 points.
